# Local IL-10 delivery modulates the immune response and enhances repair of volumetric muscle loss muscle injury

**DOI:** 10.1038/s41598-023-27981-x

**Published:** 2023-02-03

**Authors:** Tai Huynh, Cassandra Reed, Zain Blackwell, Payton Phelps, Luis C. Pinzon Herrera, Jorge Almodovar, David A. Zaharoff, Jeffrey Wolchok

**Affiliations:** 1 Department of Biomedical Engineering, College of Engineering, University of Arkansas, Fayetteville, AR USA; 2 Ralph E. Martin Department of Chemical Engineering, College of Engineering, University of Arkansas, Fayetteville, AR USA; 3 Joint Department of Biomedical Engineering, University of North Carolina-Chapel Hill & North Carolina State University, Raleigh, NC USA

**Keywords:** Interleukins, Outcomes research

## Abstract

This study was designed to test the hypothesis that in addition to repairing the architectural and cellular cues via regenerative medicine, the delivery of immune cues (immunotherapy) may be needed to enhance regeneration following volumetric muscle loss (VML) injury. We identified IL-10 signaling as a promising immunotherapeutic target. To explore the impact of targeting IL-10 signaling, tibialis anterior (TA) VML injuries were created and then treated in rats using autologous minced muscle (MM). Animals received either recombinant rat IL-10 or phosphate buffered saline (PBS) controls injections at the site of VML repair beginning 7 days post injury (DPI) and continuing every other day (4 injections total) until 14 DPI. At 56 DPI (study endpoint), significant improvements to TA contractile torque (82% of uninjured values & 170% of PBS values), TA mass, and myofiber size in response to IL-10 treatment were detected. Whole transcriptome analysis at 14 DPI revealed activation of IL-10 signaling, muscle hypertrophy, and lymphocytes signaling pathways. Expression of ST2, a regulatory T (T_reg_) cell receptor, was dramatically increased at the VML repair site in response to IL-10 treatment when compared to PBS controls. The findings suggest that the positive effect of delayed IL-10 delivery might be due to immuno-suppressive T_reg_ cell recruitment.

## Introduction

When provided the appropriate cues, skeletal muscle has a strong capacity for self-repair. Following mild muscle damage (ex. strains, contusions, and lacerations), cells are damaged but the underlying extracellular matrix (ECM) is largely intact and regeneration at the injury site is robust^1,2^. However, when significant muscle volume is lost (trauma, infection, surgical resection), or volumetric muscle loss (VML), the cues provided by the injured tissue are missing and the endogenous repair mechanisms of skeletal muscle are overwhelmed, often leading to overt fibrosis and chronic inflammation at the site of injury regeneration^3–6^. Current soft tissue repair techniques and traditional rehabilitation have not been able to reverse the pathological changes that occur following VML injury. To maximize recovery from VML injury, our group and others are exploring the use of regenerative medicine strategies as a means of restoring these lost cues. The vast majority of VML regenerative medicine research, including our own^7–12^, has focused on the development of scaffolds and muscle progenitor cells as a means to re-establish the architectural and pro-myogenic cues lost to injury. Although regenerative medicine strategies have shown tantalizing promise and warrant continued research, the results across several strategies appear to suggest that regenerative medicine alone may have a ceiling; typically restoring no more than half of the contractile force lost to VML injury^7,13–17^. What has been overlooked by us and others may be the importance of reestablishing a pro-myogenic immune environment as part of the regenerative medicine repair strategy.

The immune system coordinates muscle regeneration following injury, making it an attractive therapeutic target. What we know about muscle regeneration has been largely elucidated through the use of acute injury models (toxin, unloading/reloading, etc.). Following this type of acute injury, muscle regeneration is robust and characterized by spatially and temporally coordinated responses by leukocytes (macrophages), lymphocytes (T cells), and muscle progenitor cells (also termed satellite cells). Evidence suggests that coordination between these cells in the muscle-immune microenvironment (MIME) is mediated by soluble cytokines^18^. Recovery from acute injury begins with the activation of satellite cells that reside at the periphery of individual muscle fibers. Within hours of injury, muscle tissue is invaded by neutrophils, then later by macrophages and T-cells that are polarized toward the pro-inflammatory M1 and Th1 phenotypes, respectively. The early immune response is characterized by increased levels of pro-inflammatory cytokines, including IFN-γ, TNF-α, and IL-6, which stimulate the proliferation of satellite cells and initiate their differentiation into myoblasts^19^.

Following the initial inflammatory phase, the immune response transitions to a pro-regenerative phase, during which the available stockpile of myoblasts is recruited and further differentiated to build new muscle tissue. During this transition there is a dramatic shift in intramuscular cytokine levels from pro-inflammatory (IL-6, TNF-α, IFN-γ) to anti-inflammatory (IL-10, IL-4, IGF) cytokines^18,20^. The transition is coupled with a shift from an M1/Th1 immune cell population to one that is dominated by M2 macrophages and regulatory T cells (T_regs_)^21^. While the biology of immune cell polarization is complex and polarization is not binary, the preponderance of evidence suggests that the presence of M2 biased macrophages and Th2/T_reg_ biased T-cells within the wound site is essential for optimal muscle regeneration^18,22–24^. To be clear, the robust and well-coordinated regenerative paradigm described above was deciphered from acute injury models. While similar dynamics are expected to enhance recovery from VML repair, a detailed picture of the immune response and the MIME following VML injury is incomplete but the limited information that does exist suggests that VML injury dysregulates the regenerative response^4^. Restoring the coordinated communication between immune cells (macrophage and T cell polarization) and muscle progenitor cells (satellite cell dynamics) provides a strong rationale for the development of immunotherapeutic approaches capable of recapitulating the pro-regenerative acute injury wound healing response in order to achieve ideal clinical outcomes following VML repair. Evidence from our preliminary efforts as well as published evidence from others suggest that VML injury decreases endogenous IL-10 which is a critical cytokine produced by immune cells driving tissue repair and regeneration as well as promoting the transition from the pro-inflammatory/proliferative to the anti-inflammatory/regenerative stage of muscle healing^25–27^. The objective of this study was to determine whether exogenous delivery of IL-10 can improve the performance of a regenerative medicine strategy for the repair of VML injury.

## Results

### The IL-10 signaling node appears to be a particularly promising immunotherapeutic target for the treatment of VML injury

**Figure 1:**
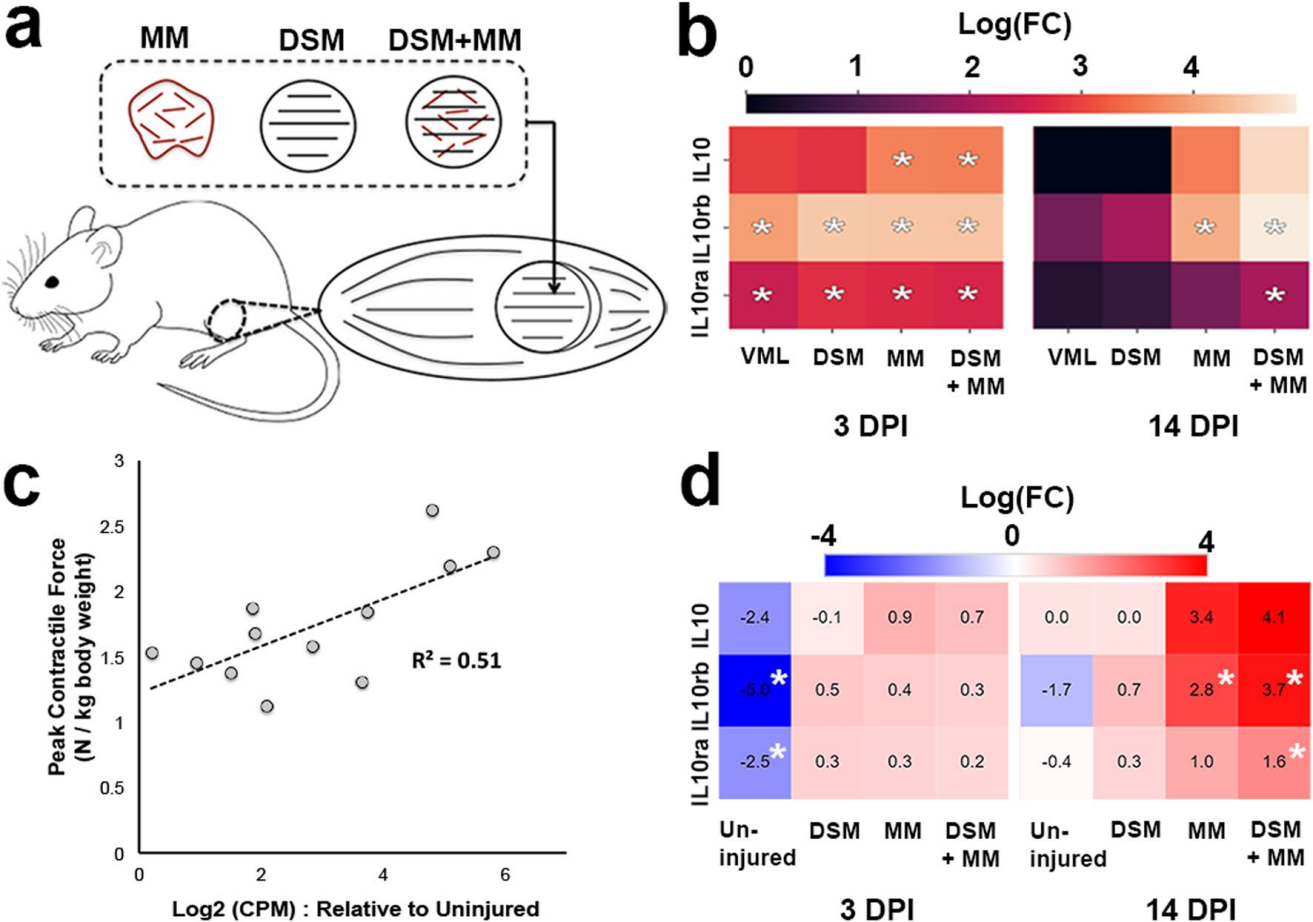
The IL-10 signaling node appears to be a promising immunotherapeutic target for the treatment of VML injury. (**a**) Rat tibialis anterior muscles were ablated with an 8 mm diameter biopsy punch to create a VML defect removing approximately 20% of muscle mass. VML defects were immediately subjected to implant repair strategies including minced muscle (MM), decellularized skeletal muscle (DSM), and a combination of the two (DSM + MM). (**b**) Heatmap of gene expression (log_2_FC) for all treatment groups relative to uninjured muscle for key IL-10 signaling related genes. (**c**) IL10R expression correlated moderately with animal limb torque outcomes at 14 DPI, indicated through linear regression with R^2^ = 0.51. (**d**) Heatmap of gene expression (log_2_FC) for all groups relative to VML no repair injured muscle for key IL-10 signaling related genes. Asterisks (*) indicate measurements meeting differential gene expression criteria; |log_2_FC|> 1.5) and p < 0.05.

We first examined and paired the early in vivo functional response induced by differing VML tissue engineering repair strategies that are broadly representative of those explored by the field (scaffold alone, cells alone, or scaffold + cells) to the transcriptomic response induced by each intervention in order to elucidate potential immunotherapeutic targets (Fig. 1a). Muscle tissue was collected from the VML repair site at 3 and 14 days post VML injury (DPI) across the differing regenerative medicine repair strategies (n = 4–5/group) and the transcriptome was examined with the aid of RNA Sequencing (RNA-Seq). Unrepaired VML injuries and uninjured normal muscle served as comparative controls. At 3 DPI, a broadly similar pattern of gene expression, dominated by upregulation of genes related to acute inflammation, was observed across all groups. However, by 14 DPI the muscle transcriptome began to vary across the differing repair strategies. Of particular interest, we observed that while expression of the IL-10 gene remained low across all groups (often undetectable) at 14 DPI, the expression of the IL-10R receptor gene was elevated in response to VML repair (Fig. 1b). The elevated expression of the IL10R gene in the VML repair groups is likely the result of an increase in the population of immune cells carrying the receptor, which as described previously are known to infiltrate muscle following injury. Furthermore, the expression of the IL10R gene was moderately correlated (R = 0.51) with early (14 DPI) muscle torque values (Fig. 1c) suggesting a link to improved functional recovery. When viewed together, the IL-10/IL10R findings point to a potential responsiveness to IL-10 signaling at the VML repair site, however the absence of significant endogenous IL-10 production at 14 DPI following regenerative medicine intervention suggests that modulation of IL-10 cytokine levels through exogenous delivery could be a promising adjunct immunotherapeutic approach. Lastly, when compared directly VML, both MM and DSM + MM groups elicited significantly higher level of IL10R transcripts (Fig. 1d). Because IL10R transcripts level from DSM + MM group were not statistically different from MM group, we decided to employ MM as the repair strategy in our IL-10 delivery experiment to reduce variability between subjects which increase our ability to detect the effect of the exogenous IL10 delivery strategy.

### Delayed IL-10 delivery improved functional and structural recovery following VML repair

**Figure 2:**
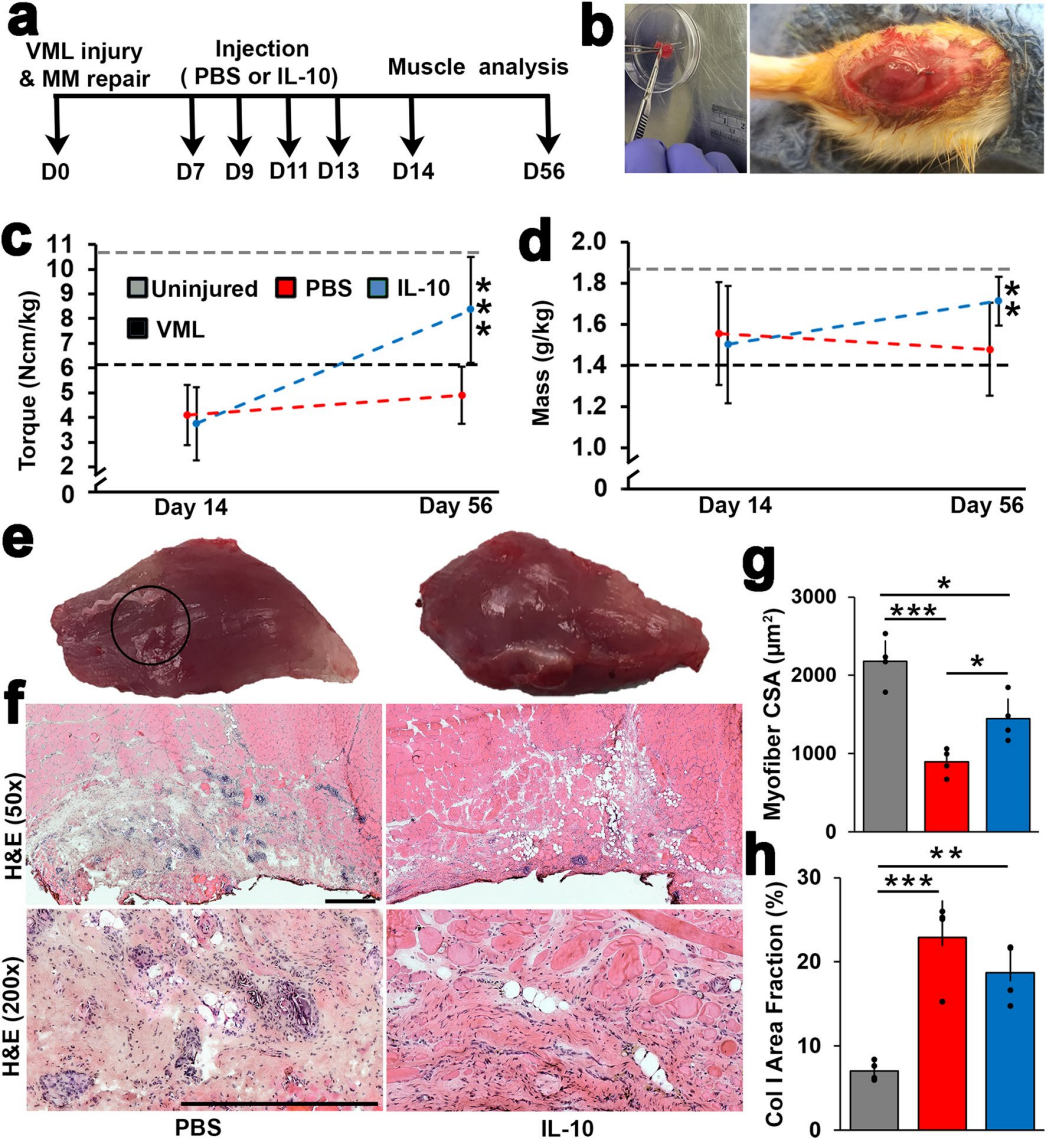
Delayed IL-10 delivery improved functional and structural recovery following VML repair. (**a**) VML injuries were created and repaired using autologous MM. One week following repair, recombinant rat IL-10 was injected at the repair site every other day for one week. (**b**) Autologous MM grafts were prepared by mincing all of the removed muscle plug using a scalpel and scissors. The resulting MM paste was packed into the defect site; the fascia at the defect site was sutured to secure the MM paste in place. (**c**) Tibialis anterior (TA) electrophysiological measurement of mean peak contractile torque (Ncm/kg rat body weight) and (**d**) mass (g/kg rat body weight) for IL-10 treated and PBS controls groups were assessed at 14 and 56 DPI. Average torque and mass from uninjured controls (accessed in this study) and VML with no repair groups at 56 DPI (accessed in previously published study^76^) were presented as 2 parallel dashed lines. Error bars are presented as ± standard deviation, with n = 5 animals per treatment group and timepoint. (**e)** Representative image of gross appearance of TA for PBS (left) and IL-10 (right) at 56 DPI are presented. Black circle indicates VML injury and repair site. (**f**) Representative H&E stained TA muscle cross-sections collected at 56 DPI from PBS and IL-10 groups at 50 × and 200 × show regions of tissue healing, cell infiltrations and the anterior TA surface. Scale bar = 250 µm. Cross-sections were quantified for (**g**) collagen I area fraction (% Col I) and (**h**) muscle fiber cross-sectional area (µm^2^). Group means + SD are presented; n = 4/group. The *, **, and *** indicate statistically significant difference of p < 0.05, p < 0.01, and p < 0.001 between groups.

To exploit IL-10 signaling sensitivity and counteract insufficient endogenous production, we next examined the impact of supplementing IL-10 cytokine at a VML repair site through exogenous delivery (200 ng/injection) delayed until one week after VML injury + repair and sustained for one week (Fig. 2a). VML injuries (removal of approximately 20% the muscle mass) were repaired using minced muscle (MM) autografts (Fig. 2b). Immediately following the IL-10 delivery period (14 DPI), mean TA muscle peak contractile torque values were similar for both the IL-10 treated (3.75 + 1.49 Nmm/kg) and PBS controls groups (4.11 + 1.22 Nmm/kg); these torques were approximately 40% of uninjured contralateral limb values (10.18 + 3.02 Nmm/kg), a statistically significant reduction in torque. However, by 56 DPI, a significant main effect of IL-10 treatment to increase peak contractile torque (p < 0.001) when compared to PBS controls was detected (Fig. 2c). Mean torque values for the IL-10 treated group (8.34 + 2.13 Nmm/kg) had returned to approximately 82% of uninjured contralateral contractile torque values (10.78 + 3.16), while 56 DPI PBS controls group values (4.90 + 1.17) had not improved from 14 DPI values. Similar to the response detected within the peak torque data, the main effect of IL-10 treatment on TA mass was at first not significant at 14 DPI but did reach significance (p = 0.002) at 56 DPI when compared to PBS controls (Fig. 2d). Specifically, IL-10 treated TA mass increased from 1.50 + 0.28 mg/g at 14 DPI post repair to 1.71 + 0.12 mg/g at 56 DPI, a recovery of 55%. At 56 DPI, IL-10 treated TA muscle mass was on average 16% larger than PBS controls group samples. Alternatively, TA mass recovery (− 21%) between 14 DPI (1.55 + 0.25 mg/g) and 56 DPI (1.48 + 0.22 mg/g) was not significant for the PBS group.

The gross morphology of whole TA muscles collected from both the IL-10 treated and PBS controls groups at 56 DPI showed evidence of repair site healing (Fig. 2e). When viewed in cross section, IL-10 treated and PBS controls repair site tissue samples (56 DPI) contained numerous muscle fibers, which were surrounded by dense connective tissue that extended proximally into the belly of the TA muscle (Fig. 2f). PBS controls repair site tissue sections were characterized by a notable presence of infiltrated cells within the connective tissue suggestive of continued inflammation in PBS group, which was not observed in IL-10 treated sections (Fig. 2f). Quantitative assessment of tissue structure revealed a significant increase (p = 0.047) in myofiber cross sectional area (CSA) of IL-10 treatment when compared to PBS controls (1695 ± 434 µm^2^ vs. 859.92 ± 176 µm^2^) (Fig. 2g), though no significant difference was detected when compared the CSA distributions of the two treatment groups (Fig. S2c). While CSA recovery was improved following IL-10 treatment, repair site fibrosis was not affected. Specifically, collagen I + area fraction (% tissue area) was statistically indistinguishable between the IL-10 treatment (18.7 ± 3.1%) and PBS controls (22.9 ± 4.4%) groups (Fig. 2h). Although not different from each other, the %NCT for both the IL-10 treated and PBS controls groups was significantly elevated when compared to uninjured contralateral controls. To summarize, while recovery was similar between the groups immediately following the 7 day delivery period (14 DPI), the delayed and sustained delivery of IL-10 significantly improved peak contractile torque (81.1 ± 17.6% vs. 45.7 ± 9.3% of uninjured controls), muscle mass (85.8 + 6.1 vs. 75 + 9.7% of uninjured controls) as well as myofiber CSA (61.2 ± 12.4% vs. 31.1 ± 2.1% of uninjured controls) recovery when compared to the PBS group at 56 DPI.

### Delayed IL-10 delivery enhanced key muscle wound healing transcripts and signaling pathways

**Figure 3:**
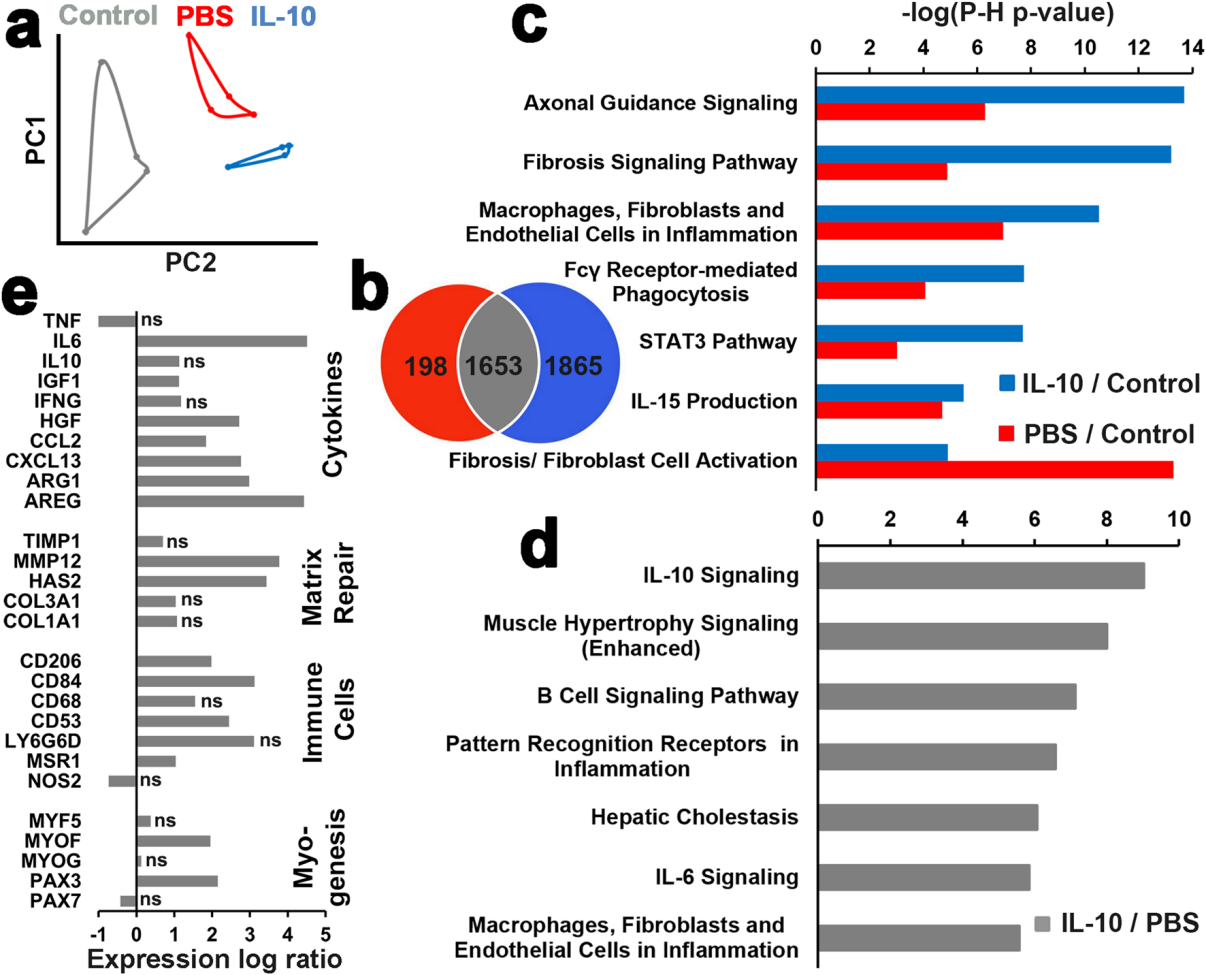
Delayed IL-10 delivery enhanced key muscle wound healing transcripts and signaling pathways. (**a**) PCA of TA skeletal muscle transcriptomes from uninjured, PBS, and IL-10 groups normalized CPM using all transcripts. (**b**) Venn diagram illustrates differentially expressed gene counts for IL-10 treatment and PBS controls groups with respect to uninjured controls. (**c**) Top canonical pathways based on the Ingenuity Pathway Analysis (IPA) assessment of differentially expressed genes (FDR < 0.05 and |log_2_FC|> 1.5) within IL-10 treatment and PBS controls with respect to uninjured controls. (**d**) Selected gene expression for key IL-10 treatment group genes with respect to PBS controls are presented as log ratio (log_2_FC). (**e**) Top canonical pathways based on IPA assessment of differentially expressed genes of IL-10 with respect to PBS. “ns” indicates no significant difference (FDR > 0.05).

We next examined the early (14 DPI) transcriptome using RNA-Seq to determine which molecular mechanisms may have led to the functional and structural improvements that occurred in response to IL-10 delivery. Principal component analysis (PCA) revealed distinct transcriptome clustering within IL-10 treated, PBS controls, and uninjured contralateral with clear separation between each group. Notably, the IL-10 treated and PBS controls groups clustered in closer proximity to one another when compared to the uninjured controls, suggesting transcriptome commonalities resulting from VML injury independent of IL-10 treatment (Fig. 3a). When normalized to uninjured controls (cut off for differential expressed genes at FDR < 0.05 and |log_2_FC|> 1.5), the two treatment groups shared 1653 differentially expressed genes (DEGs), while IL-10 had 1865 unique DEGs, and PBS had 198 unique DEGs (Fig. 3b).

Pathway analysis (normalized to uninjured controls) revealed that most of the top pathways (five pathways) were related to fibroblast/immune cell infiltration, immune cells/fibrosis signaling, and inflammation-related pathways (Fig. 3c) suggesting the delivery of exogenous IL-10 had a significant impact on immune system dynamics. It is also notable that the PBS controls group was characterized by less activation in all of the top IL-10 treatment group pathways with the exception of fibroblast cell activation. Direct comparison of the IL-10 treatment group transcriptome to PBS controls revealed that, IL-10 signaling was the most dominant pathway, as expected. Other top pathways involved enhanced muscle hypertrophy signaling, immune cell activity, and inflammation-related signaling pathways (Fig. 3d). Notably, IL-10 uniquely upregulated cell marker (CD53) present on both macrophages and myoblasts that is critical to myofiber regeneration^28^ as well as many pro and anti-inflammatory cytokines (ARG1, IL6, HGF) that were not affected in PBS controls (Fig. 3e and Fig. S1). Furthermore, IL-10 treatment enhanced expression of several chemotactic transcripts (Ccl2, Cxcl13) (Fig. 3e). These transcriptomic findings suggested that delayed IL-10 treatment broadly altered the post VML repair wound healing environment at 14 DPI which potentially enhanced muscle repair/regeneration via recruitment and activation of critical immune cell populations.

### IL-10 delivery boosted the infiltration of pro-regenerative regulatory T cells at the site of VML repair

**Figure 4:**
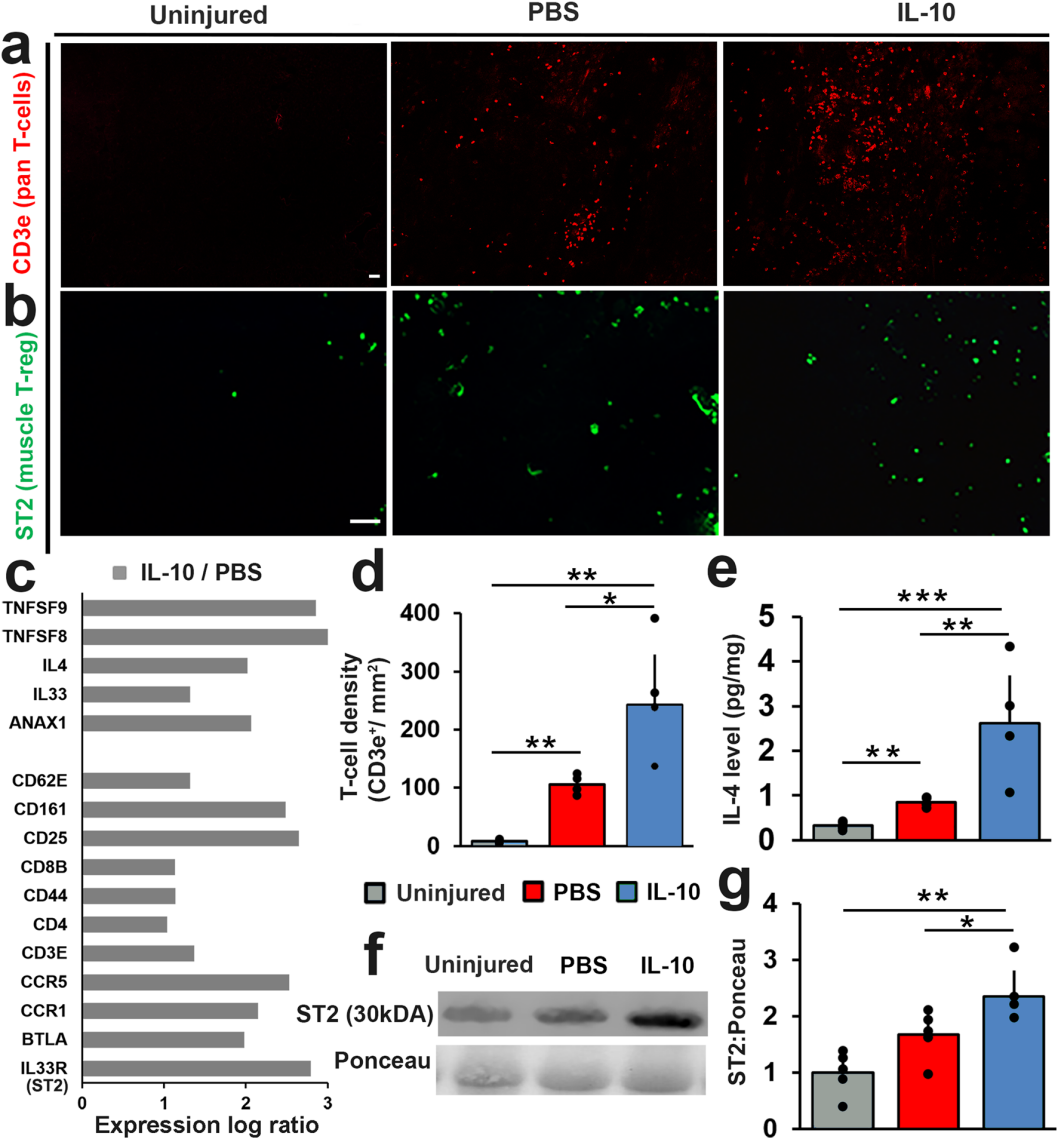
IL-10 delivery boosted the infiltration of pro-regenerative T-regulatory cells at the site of VML repair. TA muscle cross-sections were stained for (**a**) CD3e (red) and (**b**) ST2 (green). Representative TA cross-sections from uninjured, PBS, and IL-10 are presented. Scale bar = 100 µm. (**c**) Key differentially expressed (FDR < 0.05) T-cell related genes are presented as log ratio (log_2_FC) by comparing the transcriptome of IL-10 treatment to PBS controls. (**d**) CD3e immunostained tissue cross-sections were used to quantify T-cells density (CD3e^+^/mm^2^). (**e**) IL-4 concentration (pg/mg) was determined from protein lysate using multiplexed ELISA. (**f**) Representative immunoblots stained with ST2 and Ponceau (original blot images shown in **Fig. S3**). (**g**) The relative (to Ponceau) ST2 protein contents for both IL-10 treatment and PBS controls were quantified from Western blots. ST2 protein concentration values were normalized to uninjured controls. Group means + SD are presented; n = 4–5/group. The *, **, and *** indicate statistically significant difference of p < 0.05, p < 0.01, and p < 0.001 between groups.

We then examined infiltration of immune cells, using the transcriptomic data, immunohistochemistry (IHC), western blot, and multiplexed ELISA in order to narrow in on the types of immune cells present at the repair site following IL-10 treatment. At 14 DPI, immunoreactivity to the pan-T cell marker CD3e (Fig. 4a,d) was markedly increased within IL-10 treated repair site tissue, resulting in significantly (p = 0.029) higher (128% compared to PBS) T cell counts in IL10 group (242 ± 87 cells/mm^2^) tissue sections when compared to PBS controls (106 ± 15 cells/mm^2^), suggesting IL-10 treatment increased VML repair site T cell infiltration during the early post treatment phase of muscle healing. To determine if exogenous IL-10 delivery also influenced the concentration of T cell related cytokines that had previously demonstrated upregulated expression, we measured the concentration (pg/mg protein) of IL-4 & IFN-γ using the tissue lysate extracted from the defect site. We found that IL-10 treatment significantly increased the tissue concentration of IL-4 (p = 0.01), but did not affect IFN-γ. Specifically, the concentration of anti-inflammatory cytokine IL-4 measured within IL-10 treatment group tissue (2.62 ± 1.06 pg/mg) increased by 207% when compared to PBS group concentration (0.85 ± 0.09 pg/mg) (Fig. 4e). The increased IL-4 concentration and T-cell infiltration at the IL-10 treated VML repair site prompted us to examine the RNA-Seq data for unique T cell related markers. We found that IL-10 significantly upregulated the expression of several key T cell related markers (BTLA, CD4, CD8E, CD25), when compared to PBS controls, most notably IL33R/ST2, a surface receptor protein highly expressed in T_regs_ (Fig. 4c). The increased expression of ST2 within the repair site was also validated through IHC. Expression of ST2 was qualitatively increased within IL-10 treated tissue sections at 14 DPI when compared to PBS controls (Fig. 4b). Lastly, we measured the relative protein level of ST2 in all groups using western blots against ST2 (Fig. 4f). We found that while VML repair alone trended (p = 0.07) toward higher ST2 proteins level (1.65 ± 0.35) compared to uninjured controls (1 ± 0.35), IL-10 treatment significantly doubled (p = 0.047) this response (2.38 ± 0.43) (Fig. 4g). When taken together, the RNA-Seq, ELISA, western blot, and histology results provide strong evidence that IL-10 treatment had a dramatic effect on T-cell accumulation and activation, specifically coaxing the muscle environment toward an anti-inflammatory/pro-regenerative state with an elevated presence of muscle T_regs_-like cells. This finding points to a potential therapeutic mechanism responsible for the enhanced functional muscle recovery that was observed in response to delayed IL-10 treatment.

### IL-10 delivery prolonged de novo myofiber regeneration and shortened macrophage residency

**Figure 5:**
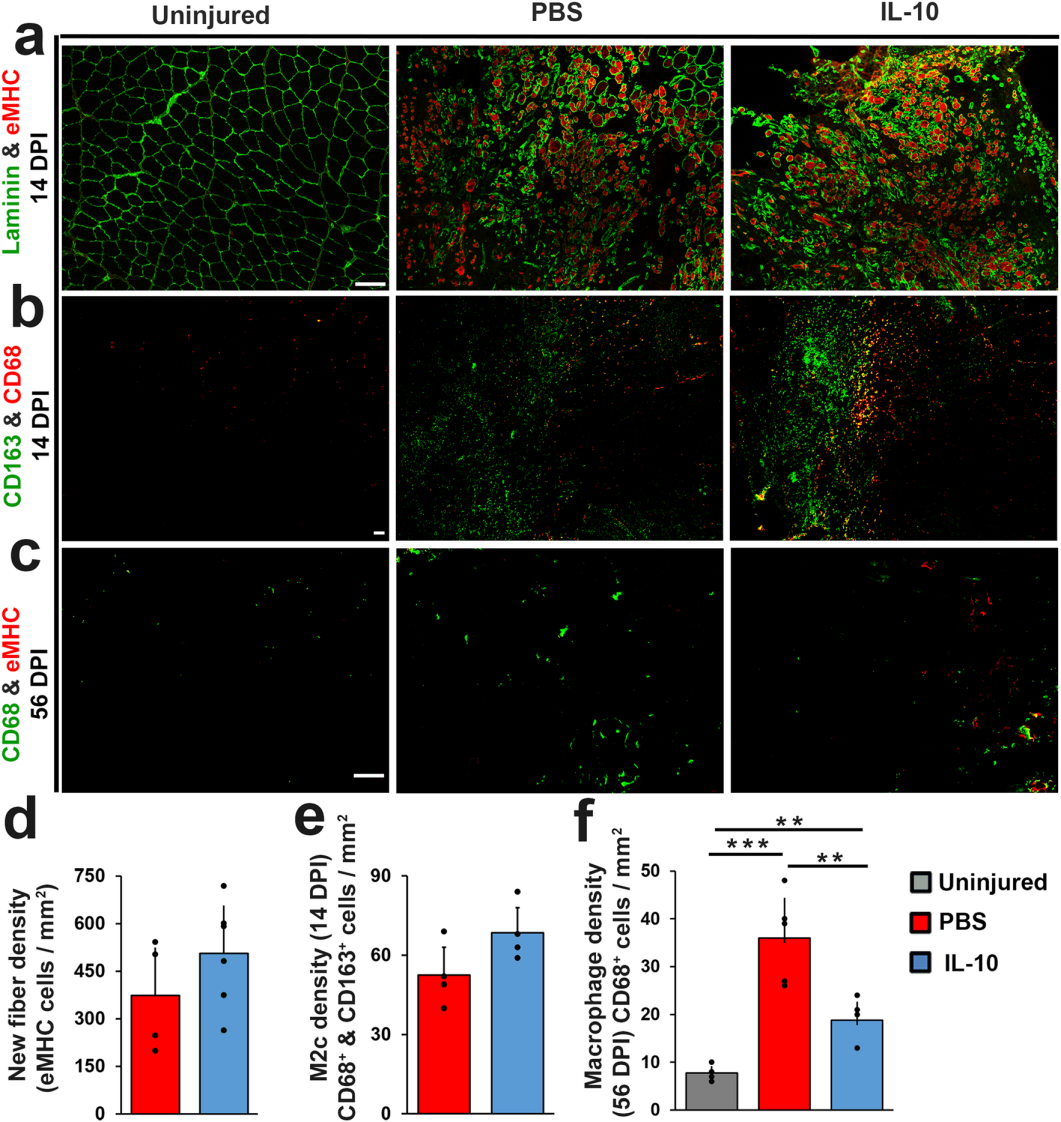
IL-10 delivery prolonged de novo myofiber regeneration and shortened macrophage residency delivery. TA muscle cross-sections prepared from 14 DPI tissue samples were co-stained for (**a**) CD68 (red) with CD163 (green) or (**b**) Laminin (green) with eMHC (red). (**c**) TA muscle cross-sections prepared from 56 DPI tissue samples were co-stained for CD68 (green) with eMHC (red). Scale bar = 100 µm. Immuno-stained tissue cross sections prepared from 14 DPI tissue samples were quantified to determine (**d**) de novo myofiber density (eMHC^+^ cells/mm^2^), (**e**) M2c density (CD68^+^ & CD163^+^ cells/mm^2^). (**f**) Immuno-stained tissue cross sections prepared from 56 DPI tissue samples were quantified to determine macrophage density (CD68^+^ cells /mm^2^). Group means + SD are presented; n = 4–5/group. The *, **, and *** indicate statistically significant difference of p < 0.05, p < 0.01, and p < 0.001 between groups.

To decipher the impact of IL-10 treatment on VML repair site myogenesis and macrophage dynamics we examined the repair site using immuno-stains directed against embryonic myosin heavy chain (eMHC), a marker protein present in de-novo myofibers, as well as the macrophage markers (CD68 and CD163) at both 14 and 56 DPI (Fig. 5a–c). VML repair site immuno-reactivity to eMHC was more widespread within the repair site following IL-10 treatment as compared to PBS controls. Although quantitative assessment of eMHC + de-novo myofiber count differences between IL-10 treatment (554 ± 116 cells/mm^2^) and PBS controls (352 ± 141 cells/mm^2^) showed some elevation in the IL-10 treatment groups, the difference approached (p = 0.058) but did not reach statistical significance (Fig. 5a,d). However, eMHC expression was sustained in the IL-10 delivery group at 56 DPI, but not in PBS controls group, suggesting that while IL-10 may not have increased de-novo regeneration immediately following the treatment period (14 DPI), it appears to prolong de-novo muscle fiber regeneration within the VML repair site tissue (Fig. 5c). Similarly, we did not detect significant differences in either pan macrophage (CD68^+^) density or activated M2c macrophage (CD163 +) density in response to IL-10 treatment at 14 DPI (Fig. 5b,e). Yet, by 56 DPI, there were significantly (p < 0.005) fewer macrophages (CD68^+^) within the IL-10 treatment group tissue sections when compared to PBS controls (Fig. 5f), suggesting that IL-10 delivery may have modulated the immune environment in the longer term, resulting in accelerated macrophage clearance.

### IL-10 delivery increased VEGF concentration and promoted repair site arteriole formation

**Figure 6:**
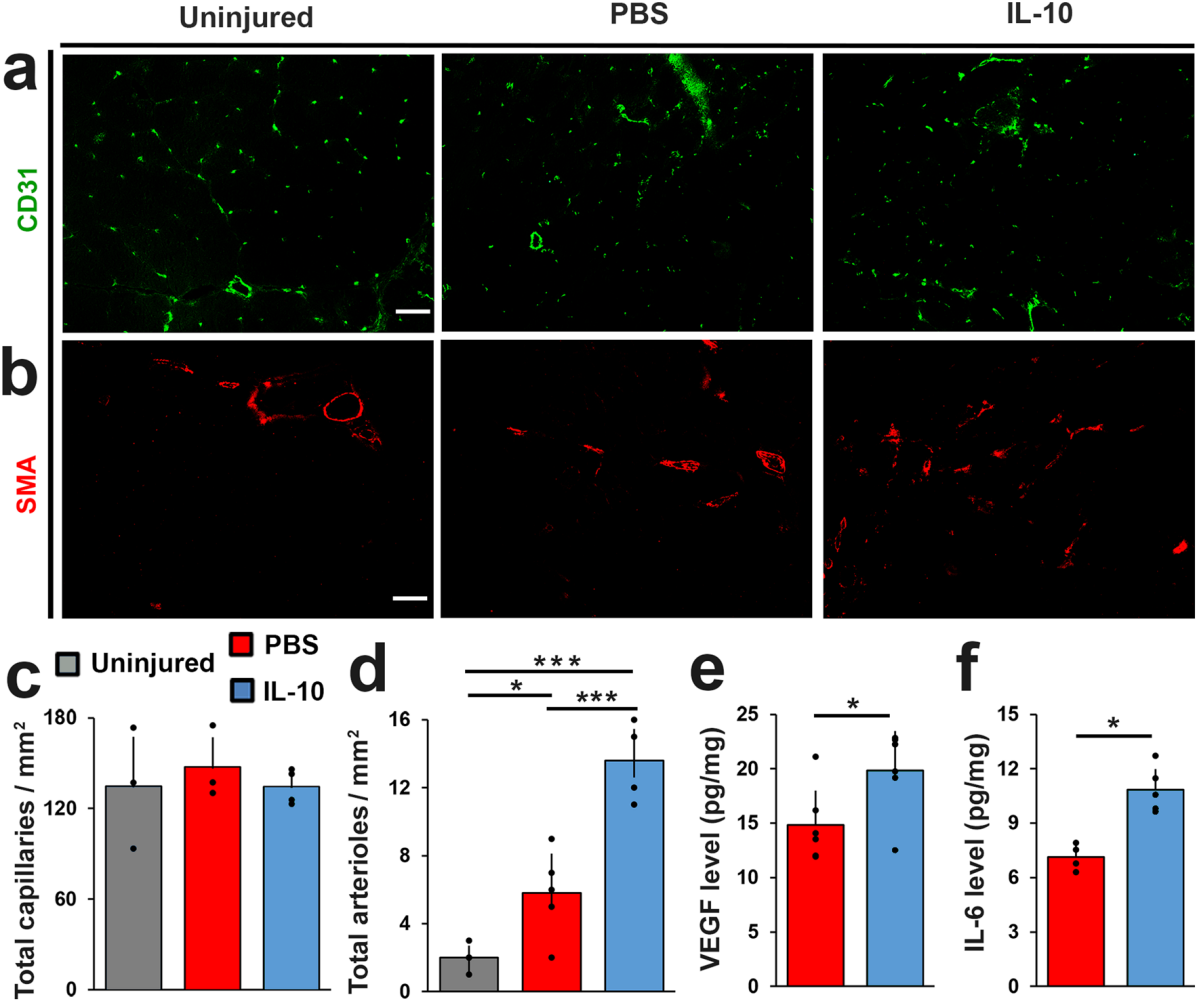
IL-10 promoted repair site arteriole formation**.** TA muscle cross-sections prepared from 56 DPI were individually stained for (**a**) CD31 (green) or (**b**) α-SMA (red). Scale bar = 100 µm. Immuno-stained tissue cross sections prepared from 56 DPI tissue samples were quantified to determine (**c**) capillary density (capillaries/mm^2^), and (**d**) arteriole density (arterioles/mm^2^). (**e**) VEGF and (**f**) IL-6 concentrations (pg/mg) were measured from 14 DPI tissue samples using multiplex ELISA. Group means + SD are presented; n = 4–5/group. The *, **, and *** indicate statistically significant difference of p < 0.05, p < 0.01, and p < 0.001 between groups.

Lastly, to assess re-vascularization at the repair site, we measured capillary and arteriole density at 56 DPI using immunofluorescent antibodies directed against CD31 and α-SMA (Fig. 6a,b). Although, we did not detect a significant difference in CD31^+^ capillary counts between groups, we did detect a dramatic (p < 0.0005) increase (150%) in arteriole density (α-SMA^+^) in response to IL-10 treatment when compared to PBS controls (Fig. 6c,d). Enhanced vascularization at the repair site via increased arteriole density could be a contributor to the sustained myofiber regeneration that was observed at 56 DPI in response to IL-10 treatment. To identify a potential trigger for the increase in arteriole density we also measured (multiplexed ELISA) the concentration (pg/mg protein) of IL-6 and VEGF, cytokines with known angiogenic signaling functions^29,30^, as well as the endothelial cell surface protein ICAM-1 using 14 DPI tissue lysate extracted from the defect site. We found that IL-10 injection significantly increased the levels of IL-6 (p = 0.001) and VEGF (p = 0.039), but did not affect ICAM-1. Specifically, the concentrations of IL-6 and VEGF collected from repair site tissue of IL-10 treatment group (0.84 ± 1.14 pg/mg IL-6, 19.87 ± 3.58 pg/mg VEGF) increased by 52% and 34% compared to PBS group (7.13 ± 0.64 pg/mg IL-6, 14.82 ± 3.16 pg/mg VEGF) respectively (Fig. 6e,f). The increase in VEGF in the absence of ICAM-1 at 14 DPI might suggest that immediately following IL-10 treatment, a nascent pro-angiogenic environment was developing that had not yet stimulated increased endothelial cell infiltration.

## Discussion

The results from this study suggest that delayed (1 week) and sustained (1 week) delivery of exogenous IL-10 can have a positive effect on muscle recovery when used in combination with regenerative medicine for the repair of VML injury. Study findings provide significant in vivo insights regarding the therapeutic potential of using immunotherapeutics to establish a pro-myogenic wound healing environment, which can in turn improve post VML repair muscle mass and contractile torque recovery. Our data are the first to show such an effect for muscle injury, and in fact, others have shown that delivery of IL-10 to injured muscle during the first week following muscle injury (days 2 and 4) slows regeneration^31^. While these prior findings contradict ours and would appear to discourage IL-10 delivery, our data underscore the importance of immunotherapy timing as well as differences in the immune response between muscle injury types (i.e., acute myotoxin injury versus VML). A full appreciation of endogenous IL-10 production would suggest that early post injury delivery is unlikely to produce much benefit and may in fact suppress the necessary pro-inflammatory phase. As we described previously, the increase in endogenous IL-10 production during tissue healing from acute non-VML injury is delayed and timed to coordinate the transition from a pro-inflammatory to an anti-inflammatory muscle microenvironment^32^. In muscle, the timing corresponds to transition from the early proliferative to the later differentiation and growth stage of myogenesis, and as such, elevated IL-10 levels may be detrimental when delivered too early or ineffective when too late. Thus, we suspect that the delayed delivery was a critical factor responsible for the enhanced recovery we observed when exogenous IL-10 delivery was paired with a VML regenerative medicine strategy. Our encouraging preliminary efforts using a delayed IL-10 delivery scheme support this hypothesis and underscore the importance of immuno-modulator delivery timing as well as the need to consider the design of injury specific immuno-modulatory strategies.

The results suggest that exogenous delivery of IL-10 enhanced VML repair/recovery, in part via its anti-inflammatory effect on both macrophage and T-cell dynamics. In particular, delayed IL-10 treatment appeared to polarize the muscle environment toward a pro-regenerative/anti-inflammatory stage of wound healing that we suspect helped sustain the formation of de novo myofibers (Fig. 5c). Alternatively, the PBS controls group was characterized by a prolonged pro-inflammatory muscle immune environment that may have inhibited the survival and maturation of de novo myofibers. Specifically, IL-10 treatment doubled T-cell counts at the VML repair site during the early post treatment period (14 DPI) and decreased macrophage counts by half at later stages (56 DPI). As such, enhancing the level of pro-regenerative cytokines like IL-10 at the site of injury is a potential two-pronged immuno-modulatory strategy as it not only plays a well recognized role in the polarization of macrophages towards the M2 phenotype^33^, but our results suggest that it also has a positive effect on T-cell recruitment. In the context of VML, this is a novel finding that indicates a potentially powerful targeting mechanism for exogenous IL-10 during muscle regeneration, and also suggests the delivery of exogenous T-cells as a complementary immuno-modulatory strategy for VML. While the endogenous delivery of single cytokines like IL-10 enables tighter control of dosage and timing, it comes with a potentially narrowed immuno-modulatory impact. Alternatively, endogenous release of numerous cytokines by T cells is temporally regulated through cellular feedback mechanisms that would be impossible to reverse engineer. As such, exogenous T-cell delivery could provide a means to modulate the production of IL-10 as well as other key immuno-modulatory factors at the wound site through manipulation of cell delivery numbers. At this stage, neither the results of this study nor literature precedents, provide a strong exclusionary rationale for the delivery of either IL-10 or T-cells, and we suggest that future head-to-head examination of both cellular and protein-based strategies could provide unique insights into performance differences between immunomodulation modalities.

The results indicate that IL-10 treatment not only increased total T-cell (CD3e^+^) numbers, but also doubled the immuno-reactivity against the ST2 protein compared to PBS^34^ (Fig. 4d,g). ST2 is a regulatory T cell (T_reg_) marker, commonly expressed by activated T_regs_ in inflammatory environments, including on T_regs_ isolated from injured muscle^35^. Recent literature suggests that the presence of T_regs_ within the wound site is beneficial to muscle regeneration (reviewed in^24^). This distinct population of ST2^+^ T_reg_ cells in skeletal muscle potentiates regeneration through direct signaling on progenitor cells and other immune cells at the injury site^36^. Specifically, the T_reg_ phenotype is known to promote muscle regeneration via the secretion of immuno-modulatory factors, including IL-4 and IL-33, that suppress pro-inflammatory M1 macrophages and Th1 cells in combination with growth factors like amphiregulin (AREG) that promote the continued expansion and maintenance of muscle satellite cells^36^. Genes for all three of these key T_reg_ products (IL-4, IL-33, AREG) were significantly upregulated in response to IL-10 treatment (Figs. 3d and 4c). Given that VML injury is known to impair the muscle’s regenerative capacity due to a prolonged and dysregulated inflammatory feedback loop that persists even following regenerative repair, the broad immunosuppressive influence of T_reg_ cells would be expected to benefit recovery. T_regs_ can further enhance wound healing through the secretion of angiogenic (VEGF) and neuro-protective (IL-35) factors that our data suggests were also upregulated in response to IL-10 treatment^37,38^. As such, increasing T_regs_ recruitment at the repair site represents an attractive mean to enhance endogenous delivery of pro-regenerative signals to the repair site that enhance the repair of multiple tissues (vessel, nerve, myofiber). To definitely establish the role of T_regs_ during recovery from VML repair, future studies by others or us could take the logical next step and probe whether the elevated T_reg_ numbers were truly beneficial using T_reg_ depleted animal models^39^. If they are, the delivery of exogenous T_reg_ cells could be exploited to enhance the performance of VML regenerative medicine.

The enhanced functional recovery in response to IL-10 treatment may also be attributed in part to improved maintenance and repair of the residual injured muscle fibers that surround the VML repair site. We found that IL-10 injections significantly increased (twofold increase) the expression of myoferlin (MYOF) (Fig. 3d). Myoferlin is highly expressed within multinucleated myofibers and surrounding mononuclear cells during muscle regeneration following injury and within myoblasts undergoing fusion during muscle development^40,41^. It has also been implicated in aiding fusion to reseal membrane disruption as well as endocytic recycling^42^. Furthermore, myoferlin can regulate growth-factor signaling, namely, promoting IL-4 production by myotubes which in turn recruit myoblasts and enhance muscle repair/regeneration^43^. Multiplexed ELISA analysis revealed that IL-10 injections more than tripled the IL-4 concentration at the muscle injury site compared to PBS controls (Fig. 4e). Because T-cells and recovering myotubes can both produce IL-4, it is difficult to determine which cell type was responsible for the increase, but we suspect that IL-10 targeted both cell populations, creating a pro-regenerative myogenic and immunogenic muscle environment. If true, our data would indicate, and the known effects of IL-10 would support^18,23^, a broad finding suggesting that IL-10 can enhance recovery from VML repair via its impact on overlapping feedback loops the modulate signaling molecules and receptors that direct the behavior of not just injury site cells, but also progenitor cells, damaged myotubes and T_reg_ cells in the surrounding muscle tissue.

We also detected an increase to both the expression (Fig. 3d) and concentration of vascular endothelial growth factor (VEGF) and IL-6 (Fig. 6e,f) at the VML repair site in response to IL-10 treatment. In the context of muscle injury, increased levels of VEGF during wound healing have been shown to enhance angiogenesis, via its effect on endothelial cell proliferation and migration^30^. As such, the increased level of VEGF in response to IL-10 treatment likely contributed to the increased arteriole density that was observed within the VML repair site at 56 DPI (Fig. 6b). The enhanced vascularization could also contribute to the prolonged de novo myofiber regeneration observed in IL-10 group. Increased VEGF concentration also plays a less recognized yet important role during axonogenesis and has been shown to accelerate neuromuscular junction regeneration, outcomes that would likely improve contractile signal transduction and in turn contractile torque recovery following VML injury and repair^44,45^. In addition to VEGF, the prolonged de novo myofiber survival observed in the IL-10 treatment group may in part be attributed to the significant increase to both the expression and concentration of IL-6 within tissues collected from the site of repair. Generally considered a pro-inflammatory cytokine^46^, the increased presence of IL-6 in response to IL-10 treatment could be viewed as paradoxical. However, IL-6 is a functionally pleiotropic growth and differentiation cytokine with context-dependent inflammatory and anti-inflammatory properties^47^. In that context, IL-6 can stimulate increased VEGF expression and production by myocytes and immune cells^48^. IL-6 can also enhance the secretion of IL-4 and IL-10 in immune cells and accelerate muscle regeneration via their effect on satellite cells^49,50^. Furthermore, studies have shown that IL-6 can enhance T cell survival and proliferation, especially under wound healing conditions, which corresponds well to our findings^51^. Although this increased IL-6 level may be responsible for the board upregulation of several chemotactic transcripts (Cxcl13, Ccl2), it was not complemented by other signs of increased pro-inflammatory cells within the IL-10 treatment group. Namely, at 14 DPI, both the IL-10 treatment and PBS controls groups had similarly high macrophage density (qualitatively), and by 56 DPI macrophage density had significantly decreased in the IL-10 treatment group (Fig. 5f). Additionally, when compared to PBS group, IL-10 did not significantly change the level of neutrophil and M1 macrophage transcripts (LY6G6D, iNOS/NOS2), but significantly increased CD206/MRC1 (pan M2 macrophage marker) HGF, and IGF1 growth factor expression (Fig. 3d), findings consistent with reduced inflammation and polarization of the muscle wound healing environment towards growth and regeneration^52,53^.

Lastly, the regenerative medicine test bed used to examine IL-10 immuno-modulatory performance deserves some discussion. We evaluated the effectiveness of a delayed IL-10 delivery strategy using autogenic minced muscle as the VML regenerative medicine strategy. Patient sourced minced muscle is a clinically attractive repair strategy that has shown regenerative potential for the repair of VML injuries by our group and others^13,54,55^. However, while we employed the minced muscle approach as the test bed, the broader goal is development of an immunotherapeutic strategy that could be widely adopted to enhance VML regenerative medicine outcomes. With that broader use in mind, we see no reason why the main finding of this study, enhancement of muscle regeneration with delayed IL-10 treatment, could not be extended to other VML strategies, including those that employ implantable scaffolds, muscle stem cells, and/or pro-myogenic rehabilitation strategies^56–59^. In order to effectively translate this strategy for broader usage, it is important to also consider that the incorporation of exogenous cytokines into a regenerative medicine strategy is not trivial as cytokines are short-lived and require exquisite spatiotemporal control for appropriate function. In the case of Il-10, I.M injection of supraphysiological concetration of this cytokine in rodent was easily cleared within a day^60,61^. The maximum IL-10 concentration used in this study was determined using previous rodent research to simulate the highest concentration range of IL-10 after muscle injury^62–65^. As for the delivery window of exogenous IL-10, there are evidence that enhanced IL-10 level as early as 3 DPI and as late as 14 DPI can be beneficial to VML recovery. Although, we delivered IL-10 at 7 DPI out to 14 DPI to establish preliminary data on IL-10 as an adjunct treatment for VML repair, future studies should expand on the optimal delivery window for this type of therapy^26,66^. Lastly, the application of IL-10 as an immunotherapeutic need not be limited to use as an adjunct in combination with regenerative medicine. In order to sustain IL-10 in this study, it was injected at the repair site every other day for one week, an apparently effective, but potentially clinically cumbersome delivery approach. Alternatively, in order to maintain exogenous cytokines at the injury/repair site, it may be more efffective to employ a sustained release approach. For example, because collagen is a major component of muscle ECM as well as many of the VML repair scaffolds being examined, it may be possible to anchor IL-10 to a scaffold and repair site using a collagen binding strategy to improve its residency. Using this technique, released IL-10 concentration can also be controlled in place of concentration spike. The indications for IL-10 delivery could include accelerated regeneration of surgical muscle trauma (ex. internal fixation placement or joint replacement) for even greater clinical impact.

## Methods

### Animal model

Adult Sprague Dawley rats (n = 20) weighing ~ 350 g, were subjected to an established VML injury model^67^. VML defects were created in the middle third of the Tibialis anterior (TA) muscle in the left leg by excising about 20% of the TA weight using an 8 mm biopsy punch. VML defect plugs were minced using a scalpel blade into 1 mm^3^ fragments and used to repair the VML injuries. Fascia and skin were closed using interrupted stiches with 5–0 Vicryl sutures (J463G, Ethicon). The contralateral limb was not injured and served as an uninjured control. Buprenorphine (0.1 mL at 0.3 mg/mL) (LIST 7571, Buprenex) was administered to all rats subcutaneously for postoperative analgesia and access to Rimadyl (MD150-2, Bio-Serv) was provided at up to 1 mg per day for seven days post-injury + repair (DPI). Food and water were provided ad libitum. All animal procedures were approved by the Institutional Animal Care and Use Committee of the University of Arkansas. All experiments were performed in accordance with all guidelines and ARRIVE guidelines. Animals were randomly assigned to one of two treatment groups (n = 10/treatment group): either rat recombinant IL-10 (400-19, PeproTech) in sterile phosphate buffered solution (PBS) (2000 ng/ml) or PBS injection (IM at site of repair) beginning 7 days after the initial surgery and continuing until day 14 (100 µL every other day = 4 injections total) (Fig. 1a,b). Animals (n = 5/treatment/time point) from each treatment group were allowed either 14 or 56 days of recovery.

### Electrophysiology

At 14 and 56 DPI, peak tetanic contractile torque of IL-10 treatment, PBS controls, and contralateral uninjured limbs was measured in vivo using techniques familiar to our groups and common to the field. To eliminate force contributions from the synergist muscle, distal tenotomies were performed on the extensor digitorum longus (EDL) and extensor halluces longus (EHL) after rats were anesthetized (2–2.5% isoflurane). Percutaneous needle electrodes were inserted into the anterior compartment of the TA to stimulate the peroneal nerve (150 Hz, 0.1 ms pulse width, 400 ms pulse train) by a S88 pulse stimulator (Grass Technologies, West Warwick) to induce contraction of the tibialis anterior muscle. Measurement of isometric torque (Nmm) was enabled by securing the foot of the limb to a force transducer system (Aurora Scientific) with surgical tape. To minimize muscle fatigue, 1-min rest periods were taken between contraction cycles. The mean isometric torque value for each limb was calculated by averaging three contractions then normalizing to total animal mass (Nmm/kg body weight). The TA and EDL muscles were then harvested, weighed, and processed for further assessments. Tissue biopsies were collected (~ 30 mg each) from the site of injury/treatment, snap frozen in liquid nitrogen, and stored at − 80 °C pending RNA isolation or protein lysate extraction. The rest of the muscle was flash frozen in isopentane (2-methylbutane) chilled in liquid nitrogen, and stored at − 80 °C. All animals were then euthanized by carbon dioxide inhalation in accordance with guidelines provided by the AVMA Panel on Euthanasia of Animals.

### Histological analysis

Tissue cross-sections (8 μm) of the repair site were obtained via cryostat (Leica BioSystems) maintained at a temperature between − 25 and − 20 °C. Prior to immunostaining, slides were permeabilized in 0.1% 100X triton and then rinsed in PBS. Sections were blocked in PBS containing 4% goat serum and 0.05% sodium azide for 1 h at room temperature. Slides were immuno-stained using primary antibodies (1:300) directed against: laminin (PA116730, Invitrogen), embryonic myosin heavy chain (eMHC) (F1.652, Hybridoma Bank), CD68 (MA6-13324, Invitrogen), CD163 (MCA342R, Bio-rad), CD3e (MA1-90,582, Invitrogen), collagen I (Col I) (NB600-450, Novus Biologicals), CD31(Pa5-32321, Invitrogen), and α-smooth muscle actin (α-SMA) (ab7817,abcam) followed by incubation of appropriate fluorescently labeled secondary antibodies (Alexa Flour, Invitrogen). Additional tissue sections were stained using hematoxylin and eosin (VWR) following manufacturer’s guidelines. All sections were digitally imaged (Nikon CiL).

Representative tissue sections collected from four to five animals per group were used for all calculations. Three separate regions/section/stain/animal were imaged (40X and 100X) at the site of injury or repair. Laminin stained images (100X) (Fig. S2b) were analyzed using in-house developed codes in ImageJ (NIH) to automatically isolate the borders of individual muscle fibers and compute muscle fiber cross-sectional area^68^. Fiber cross sectional area (µm^2^) for all fibers within an individual image (typically 100 + fibers) were calculated. Col I stained images (100X) (Fig. S2a) were analyzed using ImageJ code to isolate Col I positive tissue regions within each section and calculate the percentage of total tissue area (% Col I)^68^. Laminin co-stained with eMHC images (100X) were superimposed and to identify de-novo myofibers (fibers with clear laminin boundary and also immuno-reactive to eMHC). The total numbers of these fibers were manually counted for each imaged section and presented as de-novo myofiber density (myofibers/mm^2^). CD68, CD163 and CD3e stained images (100x) were analyzed using ImageJ code to automatically count the density of total macrophage, M2 macrophage and T-cell, respectively, presented as cells/mm^2^. CD31 and α-SMA images (100x) were manually counted to compute the density of capillary and arteriole (per mm^2^), respectively.

### Transcriptome analysis

Tissue biopsies for RNA extraction and subsequent sequencing were sent out to a commercial lab (BGI Genomic Services) for RNA-Seq analysis using BGISeq-500 platform to a mean depth of 20,000,000 reads per library. RNA sequencing reads were mapped to the Rattus norvegicus genome (RGSC build 6.0) from USCS using the 2-pass STAR protocol^69^. Reads were quantified using FeatureCounts^70^, followed by analysis of differential expression and normalization in EdgeR^71^. Differential expression was selected using a maximum false discovery rate (FDR) of 0.05 and a minimum log fold change of 1.5. Pathway level analysis was also performed using Ingenuity Pathway Analysis (IPA) (Qiagen)^72^.

### Lysate extraction

Tissue biopsies were homogenized to extract protein lysate using RIPA Lysis Buffer System (sc-24948, Santa Cruz Biotechnology)^73^. Homogenates were centrifuged at 13,000×*g* for 5 min at 4 °C, and the supernatant was collected. Protein concentration was determined using a bicinchoninic acid assay (BCA) (2322, Life Technologies).

### Multiplexed immunoassay

Magnetic Luminex Multiplex Immunoassay performed on a MAGPIX Luminex instrument and with the aid of Millipore Luminex software was used to measure the levels of various cytokines in protein lysate extracted from TA muscle tissue as previously described^74^. The Rat Premixed Multi-Analyte Kit (LXSARM-10, R&D Systems) including IL-4, IL-6, VEGF, and IL-10, ICAM-1, IFN-γ were used to create the standard curve. All reagents were prepared according to the manufacturer’s protocol provided. Each standard and sample was added in duplicate. The analysis software was set to acquire data using 50 μL of sample per well, to collect a total of 1000 beads with an average of 50 events/bead. The raw data was collected as median fluorescence intensity (MFI). The lower limit of quantification was determined using the lowest standard that was at least 3 times above background. The concentrations of each cytokine were calculated using the MFI and from the standard curve obtained. The concentrations were then normalized to the total protein in each sample, presented as pg/mg of total protein.

### Immunoblotting

Immunoblotting of proteins related to muscle protein synthesis and T-regulatory cell marker was measured as previously described^75^. Muscle homogenate (40 μg) was fractionated in 10% SDS–polyacrylamide gels. Gels were transferred to polyvinylidene difluoride (PVDF) membranes. Membranes were stained with Ponceau S before blotting to verify equal loading of the gels. Membranes were blocked in 5% milk, in Tris-buffered saline with 0.1% Tween-20 (TBST), for 2 h. Primary antibodies for 4E-BP1, Phospho-4E-BP1 (9644, 2855, Cell Signaling), and ST2 (11920-1-AP, Invitrogen) were diluted 1:1000 to 1:2000 in TBST and incubated at 4 °C overnight. Anti-rabbit and Anti-mouse monoclonal secondary antibodies (Cell Signaling Technologies) were diluted 1:2,000 in 5% milk, in TBST, and then incubated at room temperature for one hour. The Ponceau-stained membranes were digitally scanned, and the 45-kDa actin bands were quantified by densitometry and used as a protein loading correction factor for each lane. Immunoblots were imaged with the Li-Cor Odyssey Fc System and quantified with Image Studio Software (Li-Cor).

### Data analysis

Statistical analyses for assessment of non-omics data were performed on JMP software, using Student’s t-test or ANOVA with Tukey’s test post-hoc. Assessment of significance for differential gene expression was performed using adjustment of p-values (FDR) for multiple comparisons by the Benjamini–Hochberg procedure. Significance was accepted at P ≤ 0.05 (*) P ≤ 0.01 (**), P ≤ 0.001 (***), and P ≤ 0.0001 (****). Quantitative data are displayed as mean + standard deviation.

## Supplementary Information

Supplementary Figures.

## Data Availability

The datasets generated during and/or analyzed in this study are available from the corresponding author on reasonable request. All RNA-seq data generated in this study have been deposited in the National Center for Biotechnology Information (NCBI) Gene Expression Omnibus (GEO) database under the accession codes GSE206823.
