# Supplementary material for: Local IL-10 delivery modulates the immune response and enhances repair of volumetric muscle loss muscle injury
Source: Sci Rep. 2023 Feb 3;13:1983. doi: 10.1038/s41598-023-27981-x (PMC9898301; doi:10.1038/s41598-023-27981-x)
Supplement: Supplementary file 1 — Supplementary Figures. [file 41598_2023_27981_MOESM1_ESM.pdf]

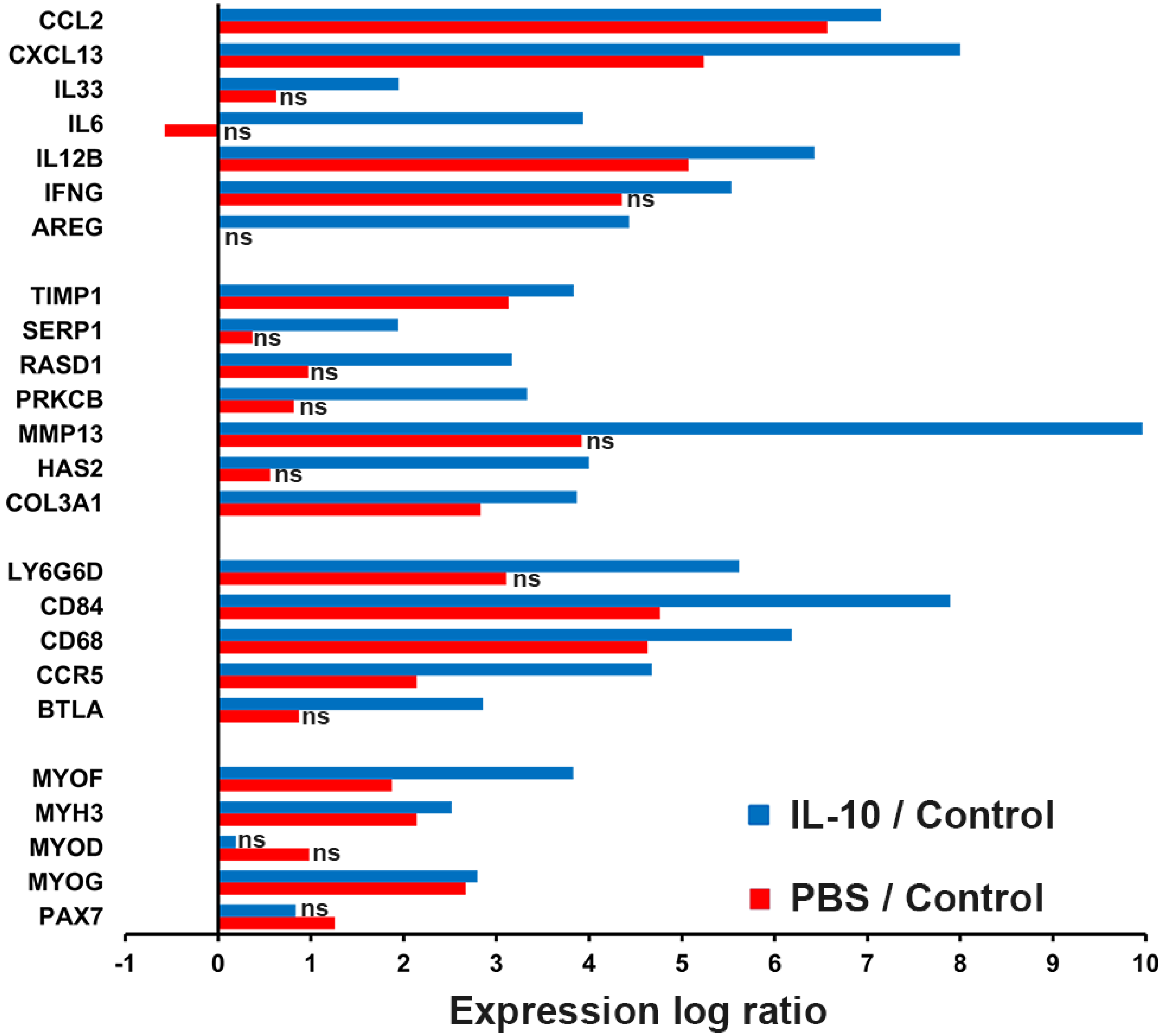

Supplementary figure 1: Selected gene expression for key IL-10 treatment and PBS controls genes with respect to uninjured controls are presented as log ratio (log_2FC). “ns” indicates no significant difference (FDR > 0.05).

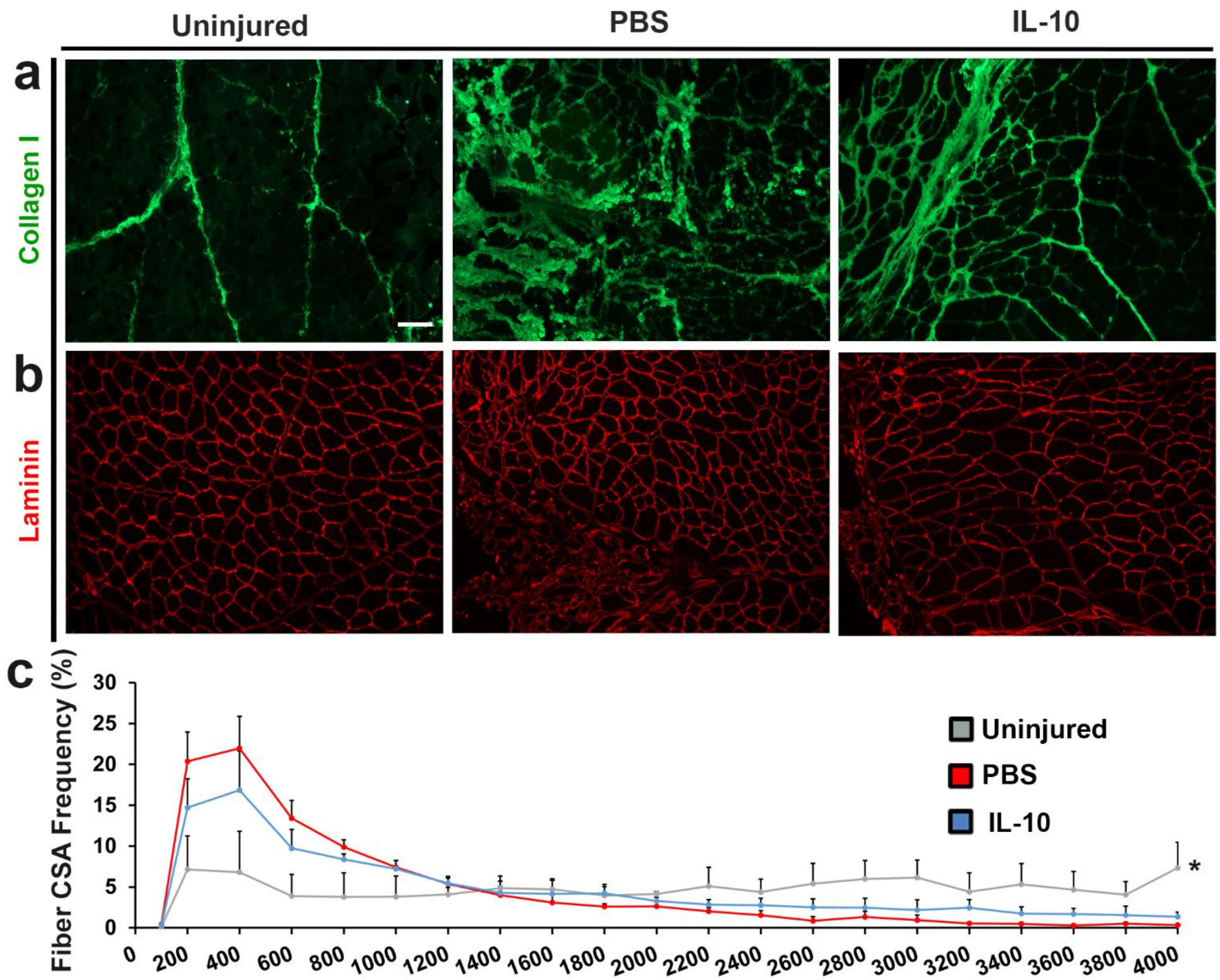

Supplementary figure 2: TA muscle cross-sections were stained for (a) collagen I (green), b) Laminin (red). Representative uninjured (left), 56 days post PBS injection (middle), and IL-10 injection (right) TA cross-sections are presented. Scale bar = 100µm. Lamin cross-sections were used to quantified for (c) muscle fiber cross-sectional area frequency distribution curve. About 600 fibers from and nearby to injury sites were included per animal, with 4 animals per groups. PBS and IL-10 curves displays a similar range of CSA values but are statistically different from injured control (Chi-squared analysis with α <0.05). Group means + SD are presented; n=4/bin/group. The * indicates statistically significant difference between uninjured controls and treatment groups.

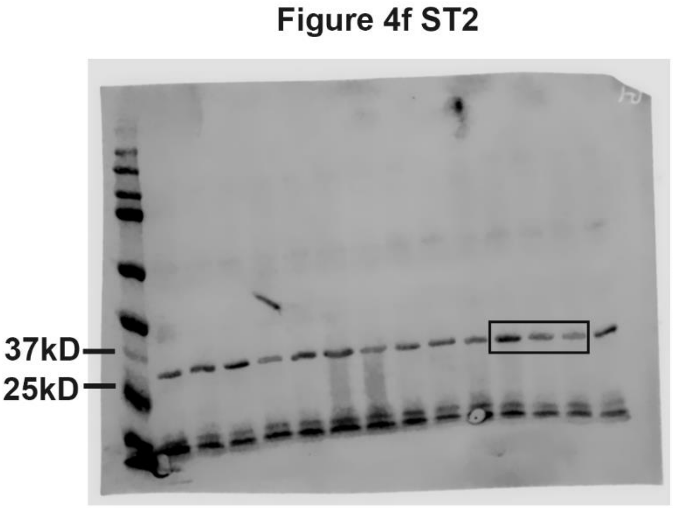

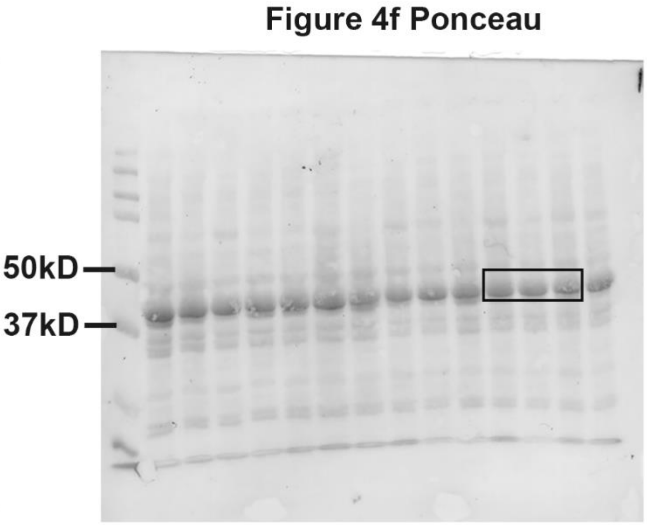

Supplementary figure 3: Original images of Western blots in **Fig. 4f**.
